# AutoWIG: automatic generation of python bindings for C++ libraries

**DOI:** 10.7717/peerj-cs.149

**Published:** 2018-04-02

**Authors:** Pierre Fernique, Christophe Pradal

**Affiliations:** EPI Virtual Plants, Inria, Montpellier, France; AGAP, CIRAD, INRA, Montpellier SupAgro, Univ Montpellier, Montpellier, France

**Keywords:** C++, Python, Automatic bindings generation

## Abstract

Most of Python and R scientific packages incorporate compiled scientific libraries to speed up the code and reuse legacy libraries. While several semi-automatic solutions exist to wrap these compiled libraries, the process of wrapping a large library is cumbersome and time consuming. In this paper, we introduce AutoWIG, a Python package that wraps automatically compiled libraries into high-level languages using LLVM/Clang technologies and the Mako templating engine. Our approach is automatic, extensible, and applies to complex C++ libraries, composed of thousands of classes or incorporating modern meta-programming constructs.

## Introduction

Many scientific libraries are written in low-level programming languages such as C and C++. Such libraries entail the usage of the traditional edit/compile/execute cycle in order to produce high-performance programs. This leads to lower computer processing time at the cost of high scientist coding time. At the opposite end of the spectrum, scripting languages such as MATLAB, Octave (John, David Bateman & Wehbring, 2014, for numerical work) Sage (The Sage Developers, 2015, for symbolic mathematics), R (R Core Team, 2014, for statistical analyses) or Python (Oliphant, 2007, for general purposes) provide an interactive framework that allows data scientists to explore their data, test new ideas, combine algorithmic approaches and evaluate their results on the fly. However, code executed in these high-level languages tends to be slower that their compiled counterpart. Due to growing interest into data science combined with hardware improvements in the last decades, such high-level programming languages have become very popular in various scientific fields. Nevertheless, to overcome performance bottleneck in these languages, most scientific packages of scripting languages incorporate compiled libraries available within the scripting language interpreter. For instance, SciPy (Jones, Oliphant & Peterson, 2014), a library for scientific computing in Python, is mainly based on routines implemented in Fortran, C and C++. To access compiled code from an interpreter, a programmer has to write a collection of special wrapper functions (aka wrappers). The role of these functions is to convert arguments and return values between the data representation in each language. Although it is affordable for a library to write a few wrappers, the task becomes tedious if the library contains a large number of functions. Moreover, the task is considerably more complex and time consuming if a library uses more advanced programming features such as pointers, arrays, classes, inheritance, templates, operators and overloaded functions. Cython (Behnel et al., 2011), Boost.Python (Abrahams & Grosse-Kunstleve, 2003), SWIG (Beazley, 2003), Rcpp (Eddelbuettel et al., 2011) and F2PY (Peterson, 2009) are considered as classical approaches for wrapping C, C++ and Fortran libraries to Python, R or other scripting languages but can only be considered as semi-automatic. In fact, while these approaches certainly ease the way of generating wrappers, the process of writing and maintaining wrappers for large libraries is still cumbersome, time consuming and not really designed for evolving libraries. Every change in the library interface implies a change in the wrapper code. Thus, developers have to synchronize two code bases that do not rely on the same kind of knowledge (i.e., C++ vs wrapper definition). To solve this issue, we provide an automatic approach for wrapping C++ libraries in Python. The critical bottleneck in the construction of an automatic approach for wrapping compiled languages libraries is the need to perform the syntactic analysis of the input code, known as parsing. Once the code has been parsed, it is possible to analyze its result for code introspection. Code introspection is the ability to examine code components to know what they represent and what are their relations to other code components (e.g., list all methods for a given class). Introspection of parsed code can therefore be used to automate the generation of wrappers.

In the past, some solutions have been developed to automate the wrapping in Python of large C++ libraries such as Py++ (Yakovenko, 2011) and XDress (Scopatz, 2013). These tools require to write *a priori* complex scripts. These scripts are then interpreted *a posteriori* to edit the code abstraction and generate wrappers. Such batch processing approaches require high-level of expertise in these software and limit the ability to supervise or debug the wrapping process. The cost of the wrapping processes with such methodologies, although automatic, is thus considered by many developers as prohibitive. The goal of AutoWIG is to overcome these shortcomings. AutoWIG proposes an interactive approach for the wrapping process and an extensible interface in Python. In particular, the proposed Python interface provides an easy-to-use environment in which the user can benefit of code introspection on large libraries. The end-user can therefore analyze compiled library components, tests different wrapping strategies and evaluates their outcomes directly.

This paper is organized as follows. ‘Requirements’ provides an insight of requirements for an automated wrapping of compiled libraries. ‘Methodology’ presents the wrapping strategies that can be considered. ‘Architecture and Implementation’ describes the main aspects of AutoWIG’s architecture and current implementations. ‘C++ Coding Guidelines’ presents C++ coding guidelines that must be respected in order to obtain the most automated wrapping workflow. ‘Results’ presents different results of AutoWIG application including in particular examples for performing partial wrapping of a library, the wrapping of template libraries and the wrapping of dependent libraries using an actual C++ statistical library set case study. In its current state, AutoWIG is limited to the wrapping of C++ compiled libraries into the high-level programming language Python using the Boost.Python C++ library. ‘Discussion’ will therefore be the occasion to discuss AutoWIG’s extensibility or limitations considering other programming languages.

## Requirements

Consider a scientist who has designed multiple C++ libraries for statistical analysis. He would like to distribute his libraries and decides to make them available in Python in order to reach a public of statisticians but also less expert scientists such as biologists. Yet, he is not interested in becoming an expert in C++/Python wrapping, even if there exists classical approaches consisting in writing wrappers with SWIG or Boost.Python. Moreover, he would have serious difficulties to maintain the wrappers, since this semi-automatic process is time consuming and error prone. Instead, he would like to automate the process of generating wrappers in sync with his evolving C++ libraries. That’s what the AutoWIG software aspires to achieve. Building such a system entails achieving some minimal features:

Type conversion management
C++ and Python have a different type system. C++ is a static language while Python is dynamic. Any wrapper needs to convert Python objects to C++, call a C++ function or method and return back to Python the C++ object or type. In AutoWIG, type conversion management is let to the wrapper system, which is Boost.Python in the current implementation. Boost.Python manages a central registry for inter-language type conversions (Abrahams & Grosse-Kunstleve, 2003). Convert methods for built-in Python types are provided by the Boost.Python library. For instance, a Python int type will be converted into its closest C++ equivalent at runtime (unsigned int, int, long, or float), but an error will be raised if the Python type is not registered and thus can not be converted to a C++ equivalent. For instance, a C++ method with an unsigned int as argument can not be called in Python with a float Python type. Moreover, subtle errors may arise when an invalid conversion method exist. As in C++, arbitrary large Python big integers will be wrongly cast into unsigned int without errors. C++ classes exposed with Boost.Python are registered as new Python type. The resulting Python object is just a wrapper around the C++ pointer of the class instance. Moreover, specific converter to standard Python type can be explicitly registered. An example is given in ‘Wrapping a template library’ for standard C++ containers. If a scientific application needs to interoperate efficiently with NumPy arrays (i.e., operate on a NumPy array without copying it) the C++ code can just link with the Boost.Python NumPy extension which defines the ndarray type in C++. The Python NumPy array will be automatically wrapped to its C++ equivalent without copy.
C++ parsing
In order to automatically expose C++ components in Python, the system requires parsing full legacy code implementing the last C++ standard. It has also to represent C++ constructs in Python, like namespaces, enumerators, enumerations, variables, functions, classes or aliases.
Pythonic interface
To respect the Python philosophy, C++ language patterns need to be consistently translated into Python. Some syntax or design patterns in C++ code are specific and need to be adapted in order to obtain a functional Python package. Note that this is particularly sensible for C++ operators (e.g., (), <, []) and corresponding Python special functions (e.g., __call__, __lt__, __getitem__, __setitem__).
Memory management
C++ libraries expose in their interfaces either raw pointers, shared pointers or references, while Python handles memory allocation and garbage collection automatically. The concepts of pointer and reference are thus not meaningful in Python. These language differences entail several problems in the memory management of C++ components into Python. A special attention is therefore required for dealing with references (&) and pointers (*) that are highly used in C++.
Error management
C++ exceptions need to be consistently managed in Python. Python does not have the necessary equipment to properly unwind the C++ stack when exceptions are thrown. It is therefore important to ensure that exceptions thrown by C++ libraries do not pass into the Python interpreter core. All C++ exceptions thrown by wrappers must therefore be translated into Python errors. Moreover, this translation must preserve the name and content of the exception in order to raise an informative Python error.
Dependency management between components
The management of multiple dependencies between C++ libraries with Python bindings is required at run-time from Python. C++ libraries tends to have dependencies. For instance the C++ Standard Template Library containers (Plauger et al., 2000) are used in many C++ libraries (e.g std::vector, std::set). For such cases, it does not seem relevant that every wrapped C++ library contains wrappers for usual STL containers (e.g., std::vector <  double >, std::set <  int >). Moreover, loading in the Python interpreter multiple compiled libraries sharing different wrappers from same C++ components could lead to serious side effects. It is therefore required that dependencies across different library bindings can be handled automatically.
Documentation
The documentation of C++ components has to be associated automatically to their corresponding Python components in order to reduce the redundancy and to keep it up-to-date.

## Methodology

A major functionality of AutoWIG is its interactivity. Interactive processing have some advantages versus batch processing. In our context, such advantages are that an interactive framework allows developers to look at the abstraction of their code, to test new wrapping strategies and to evaluate their outcomes directly. In such cases, the user must consider the following three steps:

**Parse**

In a C++ library, headers contain all declarations of usable C++ components. This step performs a syntactic and a semantic analysis of these headers to obtain a proper abstraction of available C++ components (see ‘Plugin architecture’ for details). This abstraction is a graph database within which each C++ component (namespaces, enumerators, enumerations, variables, functions, classes and aliases) used in the library are represented by a node. Edges connecting nodes in this graph database represent syntactic or semantic relation between nodes (see ‘Data model’ for details). Mandatory inputs of this workflow are headers and relevant compilation flags to conduct the C++ code parsing (see ‘Wrapping a basic library’ for an example).

**Control**

Once the Parse step has been executed, the graph database can be used to interactively introspect the C++ code. This step is particularly useful for controlling the output of the workflow. By default, AutoWIG has a set of rules for determining which C++ components to wrap, selecting the adapted memory management, identifying special classes representing exceptions or smart pointers and adapting C++ philosophy to Python (see ‘Plugin architecture’ for details). Such rules produce consistent wrapping of C++ libraries that follow precise guidelines (see ‘C++ Coding Guidelines’ for details). The Control step enables the control of parameters to ensure consistency, even if it does not fully respect AutoWIG guidelines (see ‘Wrapping a subset of a very large library’ for an example).

**Generate**

Once the control parameters have been correctly set in the Control step, the next step consists in the generation of wrapper functions for each C++ component. This is also coupled with the generation of a pythonic interface for the Python module containing the wrappers (see ‘Plugin architecture’ for details). This code generation step is based on graph database traversals and rules using C++ code introspection realizable via the graph database (e.g., parent scope, type of variables, inputs and output of functions, class bases and members). The outputs of the workflow consists in C++ files containing wrappers that need to be compiled and a Python file containing a pythonic interface for the C++ library (see ‘Wrapping a basic library’ for an example).

While an interactive workflow is very convenient for the first approaches with AutoWIG, once the wrapping strategies have been chosen, batch mode workflows are of great interest. Note that the usage of the IPython console (Perez & Granger, 2007) and its %history magic function enable to save an interactive workflow into a Python file that can be executed in batch mode using the python command line.

In some cases the compilation of wrappers can lead to some errors due to ambiguities in the internals of Boost.Python or methods of template classes that can not be instantiated on specific specializations. We developed a tool to parse compiler errors to ease the correction process of wrappers. It used mainly to either:

• Generate code that can be used in the Control step to prevent these errors in the future (e.g., classes that are not copyable by Boost.Python).
• Comment the faulty part of the code in wrappers if the error is not clearly identified (e.g., errors due to ambiguities in the internals of Boost.Python).

## Architecture and Implementation

In this section, we present the architecture of AutoWIG, describe the technical design underlying the concepts introduced in ‘Methodology’, and discuss in details the implementation choices. This section can be considered as technical and readers willing to focus first on the AutoWIG big picture can jump to ‘C++ Coding Guidelines’.

### Data model

The central data model used in AutoWIG is an abstract semantic graph (ASG) that represents code abstractions and captures code components and their relationships. In computer science, an ASG is a form of abstract syntax in which an expression of a programming language is represented by a graph whose nodes are its components (Barendregt et al., 1987). This ASG principally contains nodes identified as file-system components (e.g., directories, files) or C++ components (e.g., fundamental types, variables, functions, classes, aliases). Syntactic and semantic relations between nodes are encoded either in edges (e.g., underlying type, inherited classes), edge properties (e.g., type qualifiers, base access) or node properties (e.g., method static or const qualifications, polymorphism of a class).

### Plugin architecture

The software architecture is based on the concept of plugin (i.e., a component with a well-defined interface, that can be found dynamically and replaced by another one with the same interface). Implementations can therefore be provided by the system or from a third-party. Plugin architectures are attractive solutions for developers seeking to build applications that are modular, adaptive, and easily extensible. A plugin manager (PM) is a component in charge of discovering and loading plugins that adhere to a specific contract. As stated above, the wrapping process is decomposed into 3 steps. Each step is governed by a specific PM:

• The parser PM is in charge of the Parse step. A parser plugin implements syntactic and semantic analyses of code in order to complete an existing ASG. Its inputs are an ASG (denoted asg), a set of source code files (denoted headers), compilation flags (denoted flags) and optional parameters (denoted kwargs). It returns a modified ASG.
• The controller PM is in charge of the Control step. A controller plugin enables workflow control. It ensures that code generated in the Generate step is flawless (e.g., ensure relevant memory management, hide undefined symbols or erroneous methods of class template specializations). Its inputs are an ASG and optional named parameters. It returns a modified ASG.
• The generator PM is in charge of the Generate step. A generator plugin interprets a node subset from the ASG for code generation. Its inputs are an ASG and optional parameters. It returns in-memory files (denoted wrappers) whose content corresponds to the generated code.

Considering these PMs, the workflow simply consists in passing the ASG step by step. Plugin implementation requires different levels of expertise (see Table 1). However, the registration of a new plugin in AutoWIG is simple due to the usage of the entry points mechanism provided by the Setuptools Python package. Moreover, the concept of AutoWIG plugin manager enables an easy control of plugin implementation (see ‘Wrapping a template library’ for an example).

**Table 1:** Plugin architecture of AutoWIG. Each step of the AutoWIG wrapping workflow is managed by a plugin manager that enables an easy control of the workflow outputs. Considering the finality and underlying complexity of these plugins, implementations responsibilities are shared between AutoWIG developers and end-users. The parser and generator plugins are respectively concerned with compiled and scripting languages admissible bindings. Since such implementations require a high-level of expertise and a variety of tests, they mostly concern AutoWIG developers. On the contrary, controller plugins are library dependent and only require the manipulation of the abstract semantic graph via Python code. Thus, most of AutoWIG end-users are concerned with controller implementations.

Workflow step	Manager	Plugin implementation	Finality
Parse	parser	Developer	Performs syntactic and semantic analysis of input code and produces an abstract semantic graph
Control	controller	End-user	Regroups Python code editing the abstract semantic graph for workflow control.
Generate	generator	Developer	Traverses the abstract semantic graph and generates code given code generation rules.

**Parsers** Currently, AutoWIG provides one parser for C++ libraries. Parsing C++ is very challenging and mainly solved by compiler front-ends (Guntli, 2011) that generate abstract syntax trees (ASTs). There are many benefits in using a compiler front-end for parsing C++ code. In particular, the parser implementation simply uses the compiler front-end for performing syntactic and semantic analyses of code rather than performing itself a custom analysis of an evolving and complex language. Therefore, the implementation mainly consists in AST traversals to complete ASGs, which is a far less challenging problem. Since the development of LLVM (Lattner & Adve, 2004) and Clang (Lattner, 2008) technologies, the AST, used for the compilation process, is directly available in Python via the libclang Python package.

Our libclang parser was therefore designed using libclang:

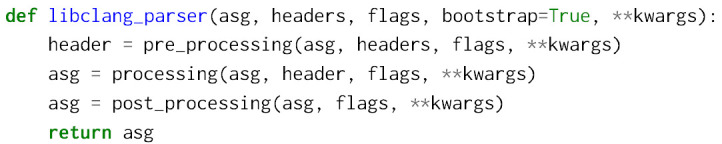



This implementation consists in the three following steps:

**Pre-process**

During the pre_processing step, header files (headers) are added in the ASG and marked as self-contained headers (see ‘C++ Coding Guidelines’ for details). Note that in order to distinguish headers of the current library from headers of external libraries that are included by these headers, the headers of the library are marked as internal dependency headers (opposed to external dependency headers). This step returns a temporary header (header) that includes all given headers. This approach enables to parse only one header including all others and therefore prevents the multiple and redundant parsing of headers. Note that compilation flags (flags) are also parsed in order to save C++ search paths (given by the -I option).

**Process**

During the processing step, the actual C++ code is parsed using the libclang Python package. The parsing of the temporary header (header) returns an AST. The ASG is updated from the AST by a process of enrichment and abstraction. The enrichment entails the addition of node properties (e.g., if a class can be instantiated or copied, if a method is overloaded) or edges (e.g., forward-declarations, back-pointers to base classes, type of variables). The abstraction entails the removal of details which are relevant only in parsing, not for semantics (e.g., multiple opening and closing of namespaces).

**Post-process**

During the post_processing step, the C++ code is bootstrapped. Template class specializations are sometimes only declared but not defined (e.g., a template class specialization only used as a return type of a method). In order to have access to all the definitions of template class specialization, AutoWIG parses a virtual program of undefined template class specialization definitions (e.g., using sizeof(std::vector<  int >); for forcing std::vector<  int > definition). Note that this step induces new undefined template class specializations and must therefore be repeated until no more undefined template class specializations arise. This recursion step is controlled by the bootstrap parameter that can be set to True, False or an integer corresponding to the maximal number of repetition of this operation (True is equivalent to bootstrap=float(”inf”) and False to bootstrap=0).

**Controllers** By default, AutoWIG provides a controller for libraries respecting some recommended guidelines (see ‘C++ Coding Guidelines’ for details):

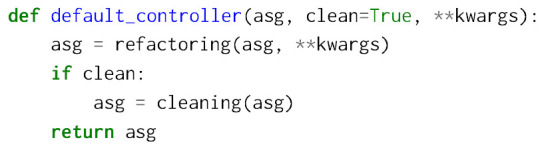



This default implementation consists of the two following steps:

**Refactoring**

The refactoring of the C++ code is simulated in order to have wrappers compliant with Python rules. In C++, some operators (e.g., operator+) can be defined at the class scope or at the global scope. But in Python, special methods corresponding to these operators (e.g., __add__) must be defined at the class scope. Therefore during refactoring, all operators, that are defined at the global scope but could be defined at the class scope, are moved as a method of this class.

**Cleaning**

The cleaning operation removes useless nodes and edges in the ASG. A library often depends on external libraries and headers. There are therefore a lot of C++ components, defined by external headers, that are not instantiated and used by the C++ code of the actual library. First, in order to remove only these useless nodes, all nodes are marked as *removable*. Then, nodes defined by the internal library are marked as *non-removable*. Recursively, all dependencies of nodes marked as non-removable are marked as *non-removable*. Finally, all nodes still marked as *removable* are removed from the ASG. Some C++ libraries, such as armadillo (Sanderson, 2010), provide one self-contained header that only includes all library headers. In such cases all C++ components will be marked as external dependency and the clean parameter of the default controller should be set to False. Otherwise, without any instruction, all these C++ components would be removed.

As soon as a C++ library does not respect the recommended guidelines of AutoWIG, the end-user has to implement a controller. As stated above, this controller will ensure that code generated by the Generate step is flawless. This step mostly consists in the addition of information concerning memory management, undefined symbols and erroneous methods of class template specializations or undesired C++ components in Python (see ‘Wrapping a subset of a very large library’ for an example).

**Generators** AutoWIG provides one generator for wrapping C++ libraries using the Boost.Python library. AutoWIG could generate wrappers in the C interface that extend the Python interpreter, but this low-level approach does not provide the abstraction needed to consider the requirements presented in ‘Requirements’. Thus, there are many benefits in using one of the semi-automatic approaches (e.g., Boost.Python, SWIG) within wrappers code.

In particular, AutoWIG uses the Boost.Python library to propose:

• An automatic Python documentation using C++ documentation since documentation strings can be injected directly in wrappers.
• A consistent adaptation of C++ patterns to Python thanks to globally registered type coercions, possible manipulation of Python objects in C++, and an efficient overloaded function handling.
• A consistent memory management thanks to the definition of call policies which can be used to handle references and pointers.
• An automatic translation of C++ exceptions into Python errors using C++ exceptions handling and conversion into Python errors.
• An automatic management of dependencies thanks to automatic cross-module type conversions.
• The possibility to inherit from C++ classes within Python and to override virtual methods.

The boost_python generator was therefore designed to generate Boost.Python wrappers:

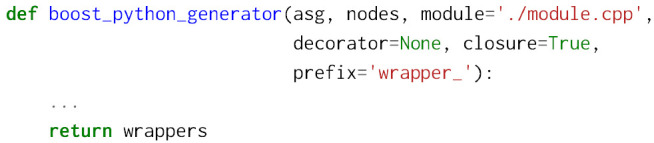



Boost.Python extensively uses C++ class templates. However, class templates may use a huge amount of memory that can entail compilation problems. To avoid this kind of problems, our implementation mainly consists in dispatching wrapper code for C++ components (nodes) into different files:

Module file
A module file is created in the ASG and named according to the module parameter. This module file is associated with multiple export files (see below). Its content corresponds to the inclusion of wrappers defined in their associated export files within a BOOST_PYTHON_MODULE block. The compilation of this file produces a Python library containing all the C++ wrapped components. This library has the same basename as the module file prefixed by an underscore.
Export files
Export files are created in the ASG within the same directory as the module file. Their content declares Boost.Python wrappers for associated C++ components. The export file of a C++ component is named by the concatenation of its prefix parameter and an unique identifier (an hexadecimal number computed from the global name of the component). As a consequence, AutoWIG creates as many files as namespaces, enumerators, variables, bunch of overloaded functions and classes given in the nodes parameter. Note that enumerators, fields and methods wrappers are included in their parent scope export file. Moreover, in order to prevent name collisions in Python, C++ components are wrapped in Python modules corresponding to their C++ scope.
Decorator file
A decorator file, named according to the decorator parameter, is created in the ASG (if decorator is not set to None). The Boost.Python library does not provide a way to wrap aliases. Moreover, for serialization purposes, member (i.e., class scoped declarations) classes or enumerations must not be wrapped as class member but as module member. The decorator of the Python code defines aliases or produces member aliases for member classes or enumerations. Note that, in some cases, programmers want to decorate the C++ like interface into a more common Python interface. For this purpose, the decorator contains lists grouping, for a template class, all its instantiations. This allows to easily select all these instantiations in order to decorate them in the same way.

The code written in each of these files is generated using the Mako templating engine (Bayer, 2012). Template engines are classically used in Web frameworks to generate dynamic HTML pages. In our case, we use a template language to generate automatically C++ wrapper code from patterns found in the ASG. Changing code generation would require only to change the template code. In order to provide a modular wrapper generation, templates must be encapsulated into classes. Class selection for the previous files is governed by plugin managers (see Table 2).

**Table 2:** Plugin managers to control the boost_python generator. Three plugin managers are used in the boost_python generator. This enables the choice of Mako templates (Bayer, 2012) to compute the content of wrappers. The generation of wrappers is therefore customizable.

Plugin	
Manager	Finality
boost_python_export	Returning a class containing templates for the generation of Boost.Python wrappers for C++ components.
boost_python_module	Returning a class containing templates for the generation of Boost.Python module for Boost.Python wrappers.
boost_python_decorator	Returning a class containing templates for the generation of Python code to complete Boost.Python wrappers.

If the parameter closure is set to True, all the dependencies of the input C++ components (nodes) are also wrapped, unless they are explicitly marked as non-exportable. To mark a node as non-exportable, its boost_python_export property has to be set to False (see ‘Wrapping a subset of a very large library’ for an example). Note that the boost_python generator does not respect the contract of generator plugins since it requires asg and nodes as inputs, in place of requiring only asg. In fact, this implementation is used in all other implementations of generator that only needs to define abstract semantic graph (asg) traversals to compute nodes that will be considered as inputs of the boost_python generator:

• The boost_python_internal generator selects all the nodes that are declared in headers marked as internal dependency headers.
• boost_python_pattern generator selects all nodes that match a regular expression denoted by the pattern parameter. This pattern parameter is set by default to ”.*”, so all the nodes are considered.

## C++ Coding Guidelines

Considering the requirements presented in ‘Requirements’, we recommend to use the following guidelines in order to benefit from the most automated wrapping procedure.

**Parse self-contained headers** An AutoWIG parser requires self-contained headers. In other words, a header should have header guards, should include all other headers it needs, and should not require any particular symbols to be defined.

**Use smart pointers** Let us consider a C++ template function declaration that returns a pointer,

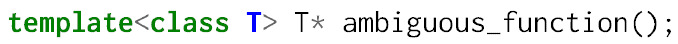



There is *a priori* no way to know whether the pointer should be deleted or not by the caller. Boost and STL (Standard Template Library) libraries have introduced smart pointers as a design pattern to ensure correct memory management. Smart pointers (i.e., unique_ptr, shared_ptr and weak_ptr) define how to manage the memory of a pointer, take the responsibility to delete the pointer, and thus remove these C++ ambiguities. In the following example,

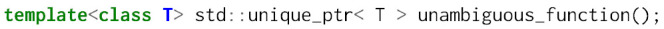



the usage of std::unique_ptr explicits the fact that the caller takes ownership of the result, and the C++ runtime ensures that the memory for T* will be reclaimed automatically. By default, AutoWIG considers that any raw pointer should not be deleted by the caller. If this is not the case, Boost.Python call policies can be set to ensure proper memory management.

**Use C++ STL containers** In C++, containers can be expressed as C arrays (e.g., int array[10];) or pointers to arrays (int* ptrarray = array;). However, C++ components (e.g., variables, functions) that are using C arrays or pointers to arrays are not wrapped by the boost_python generator due to ambiguity (if the user tries to force the wrapping, an error is raised). In these cases, we recommend to use C++ arrays (e.g., std::array<  int, 10 >) or dynamic arrays (e.g., std::vector<  int >), which can be effectively wrapped using the boost_python generator.

**Derive from std::exception** In C++, exceptions provide a way to react to exceptional circumstances in programs, like runtime errors, by transferring control to special functions called handlers. The C++ standard library provides a base class—std::exception defined in the <exception> header—especially designed to declare objects to be thrown as exceptions. By default, for a Python interfaced C++ library, Boost.Python translates a C++ exception thrown by wrapped functions or module into a Python RuntimeError. To produce better error messages, AutoWIG ensures that any exception derived from the std::exception class is correctly translated (i.e., the error raised has the same class name and content).

**Figure 1:**
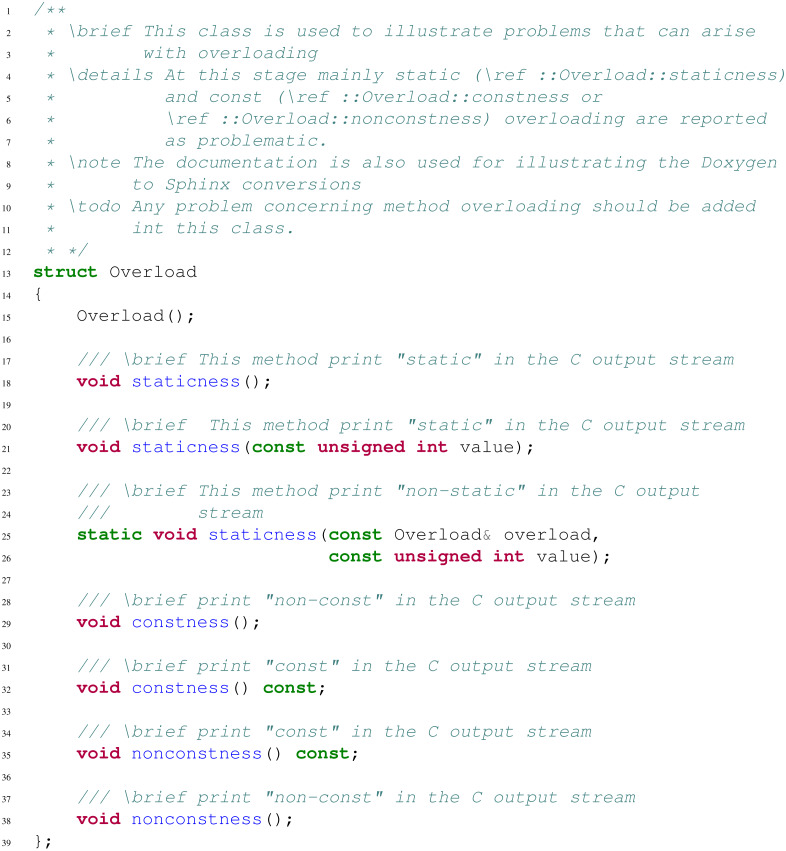
A basic header used for illustrating overloading problems. The method void staticness(const unsigned int value) (resp. void constness() or void nonconstness() const) can be wrapped but as soon as static void staticness(const Overload& overload, const unsigned int value) (resp. void constness() const or void nonconstness()) is also wrapped, it will not be callable in the Python interpreter.

**Pay attention to static and const overloading** Let us consider the header presented in Fig. 1. We here assume that the library has been wrapped using AutoWIG in a basic Python package.

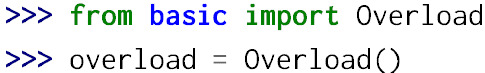



Python is not designed for function overloading but Boost.Python provides some meta-programming mechanisms in order to perform dispatching and therefore enable function overloading in Python. Yet, considering static and const specifiers, a few problems can arise:

• Overloading a function with static renders all overloaded methods as static methods. If this entails strange usage of methods that are actually not static, it remains possible to call all overloaded methods. 
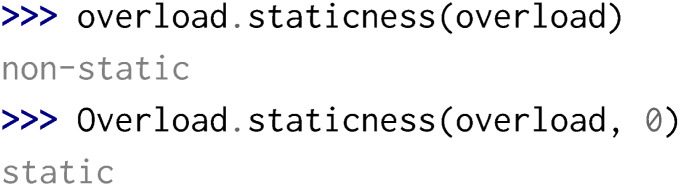
 Yet, if the static overload has for its first parameter an instance, a reference or a pointer to its parent class and all the following parameters correspond to another non-static overload, the non-static method will not be callable in the Python interpreter. 
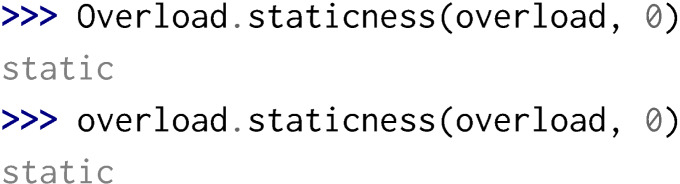

• Overloading a function with const hides the previous one written in the header. 
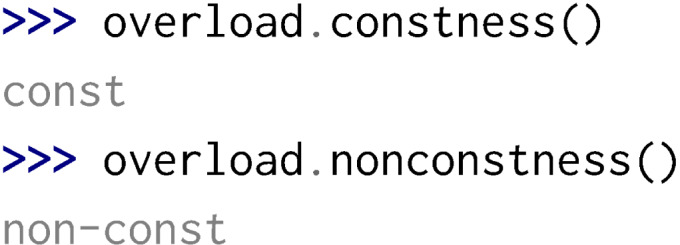
 This can have serious side effects on the library usage. We therefore recommend to specify in the controller implementation which overload must not be considered, or to design headers considering this rule.

**Use namespaces** Namespaces prevent name conflicts in large projects. Symbols declared inside a namespace block are placed in a named scope that prevents them from being mistaken for identically-named symbols in other scopes. The usage of a base namespace for each C++ library (e.g., std, boost) is highly recommended since it eases code introspection with AutoWIG.

**Document with Doxygen and Sphinx** For C++ documentation, Doxygen (Van Heesch, 2008) is one of the most standard tool for generating formatted, browsable, and printable documentation from annotated sources. Its equivalent for Python is Sphinx (Brandl, 2009). Writing and verifying documentation is a fastidious task, and the redundancy between C++ and Python wrapped components must be limited. As illustrated below, AutoWIG parses the Doxygen documentation in the C++ code source (see Fig. 1) and formats it into a Sphinx documentation. This documentation string is then injected into the Python components.

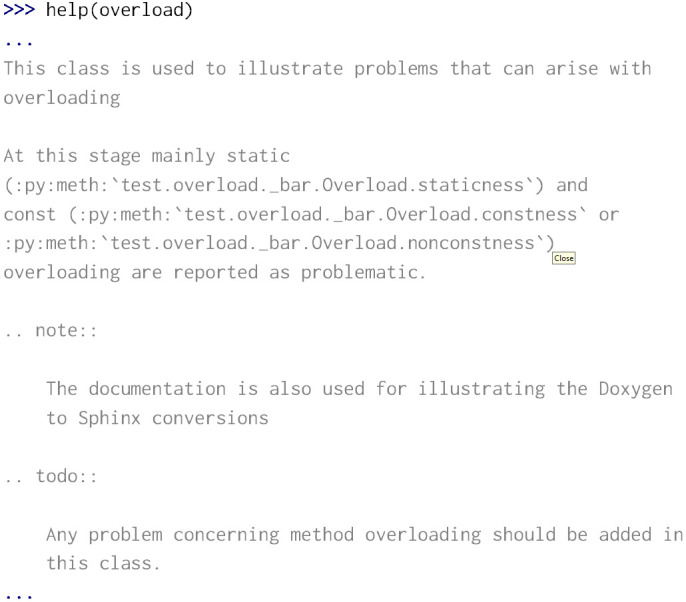



## Results

In the following section, we present some examples using AutoWIG in order to emphasize particular aspects of the wrapping process. Therefore, most of the presented examples are truncated or modified for the sake of clarity and simplicity. Nevertheless, these examples are all fully available and reproducible on a Jupyter notebook server (see ‘Installation and usage’ and supplementary materials for details).

### Wrapping a basic library

**Figure 2:**
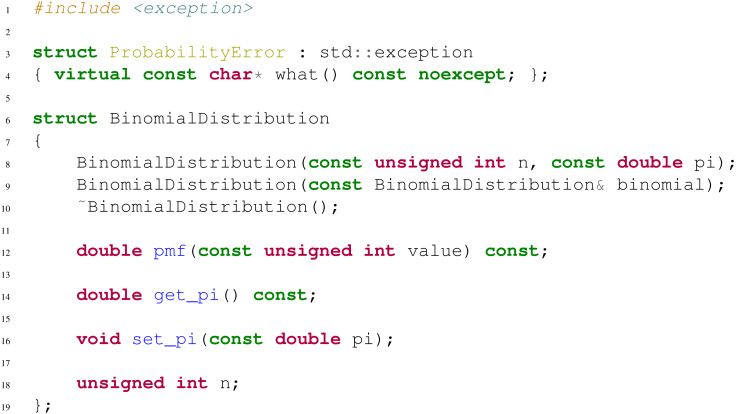
A header for a basic library. This basic C++ library implements probability mass function computation for binomial distributions (BinomialDistribution::pmf). If a user tries to set the probability parameter of the binomial distribution (BinomialDistribution::_pi) to values outside the interval [0, 1], a ProbabilityError exception is thrown.

In this example, we present the interactive wrapping workflow. For the sake of simplicity, we consider a basic example of C++ library (see header presented in Fig. 2).

First, import AutoWIG.

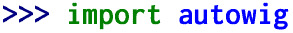



Assuming that the header is located at ‘/basic/binomial.h’, we parse it with relevant compilation flags.

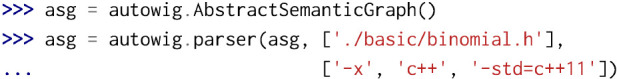



Since most of the AutoWIG guidelines are respected, the default controller implementation is suitable.

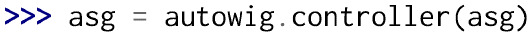



In order to wrap the library, we need to select the boost_python_internal generator implementation.

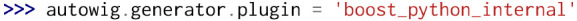



The Boost.Python module name chosen is ‘/basic/module.cpp’. Similarly, the Boost.Python decorator name chosen is ‘/basic/_module.py’.

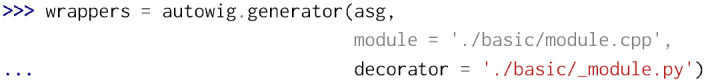



The wrappers are only generated in-memory. We therefore need to write them on the disk to complete the process.

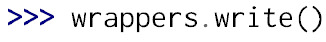



Once the wrappers are written on disk, we need to compile and install the Python bindings. Finally, we can use the C++ library in the Python interpreter:

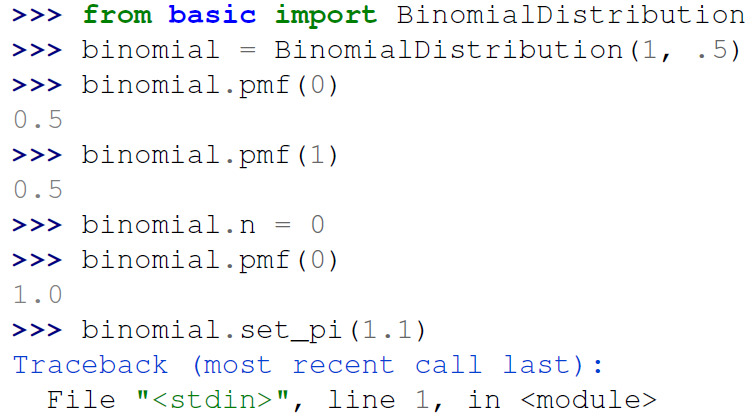



### Wrapping a subset of a very large library

Sometimes, for a very large library, only a subset of available C++ components is useful for end-users. Wrapping such libraries therefore requires AutoWIG to be able to consider only a subset of the C++ components during the Generate step. The Clang library is a complete C/C++ compiler. Clang is a great tool, but its stable Python interface (i.e., libclang) is lacking some useful features that are needed by AutoWIG. In particular, class template specializations are not available in the abstract syntax tree. Fortunately, most of the classes that are needed during the traversal of the C++ abstract syntax tree are not template specializations. We therefore bootstrapped the Clang Python bindings using the libclang parser of AutoWIG. This new Clang Python interface is called ClangLite and is able to parse class template specializations (see supplementary materials). As for libclang, this interface is proposed only for a subset of the Clang library sufficient enough for proposing the new clanglite parser.

In order to wrap a library subset, the user need to define a controller implementation that specifies which C++ components will be considered during the Generate step. The controller implemented is the following:

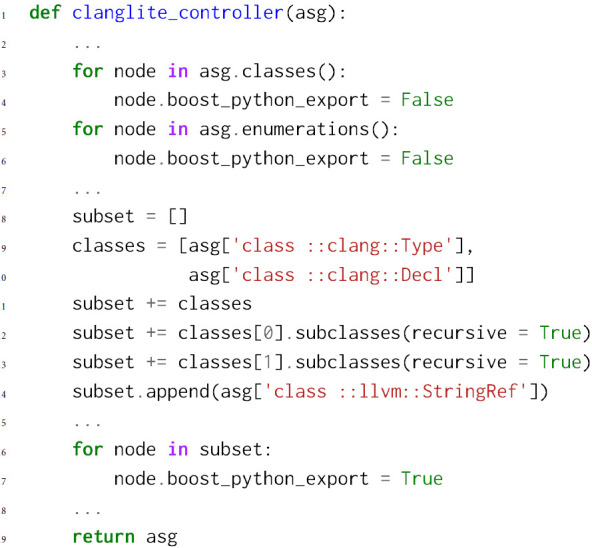



This clanglite controller principally consists in:

• Considering all user-defined types as non-exportable. This is done by setting the property boost_python_export of classes and enumerations to False (lines 3–6).
• Considering a subset of all user-defined types as exportable. This is done by first selecting the C++ components of interest (subset) using code introspection (lines 8–14). Then, the boost_python_export property of all subset components is set to True (lines 16–17).

Assuming that the asg already contains all C++ components from the Clang library and that the clanglite_controller has been defined in the Python interpreter, we need to register the clanglite_controller as a controller implementation and then to select it.

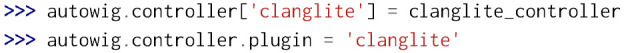



After the generation and compilation of wrappers (using the same procedure as the one described in ‘Wrapping a basic library’), it enabled us to propose a new parser implementation called clanglite. This has been done by writing Python code responsible for the traversal of the AST and the completion of an existing ASG. Contrary to the libclang parser, the AST traversed by the clanglite parser contains template classes and their specializations. This parser is therefore more efficient and is selected by default in AutoWIG, as soon as the ClangLite bindings are installed.

### Wrapping a template library

A template library is a library where there are only template classes that can be instantiated. Wrapping such libraries therefore requires AutoWIG to be able to consider various C++ template classes instantiations during the Parse step. The Standard Template Library (STL) library (Plauger et al., 2000) is a C++ library that provides a set of common C++ template classes such as containers and associative arrays. These classes can be used with any built-in or user-defined type that supports some elementary operations (e.g., copying, assignment). It is divided in four components called algorithms, containers, functional and iterators. STL containers (e.g., std::vector, std::set) are used in many C++ libraries. In such a case, it does not seem relevant that every wrapped C++ library contains wrappers for usual STL containers (e.g., std::vector<  double >, std::set<  int >). We therefore proposed Python bindings for some sequence containers (e.g., vector of the std namespace) and associative containers (e.g., set, unordered_set of the std namespace). These template instantiations are done for various C++ fundamental types (e.g., int , unsigned long int, double) and the string of the std namespace. For ordered associative containers only the std::less comparator was used.

In order to wrap a template library, the user needs to write headers containing aliases for desired template class instantiations:

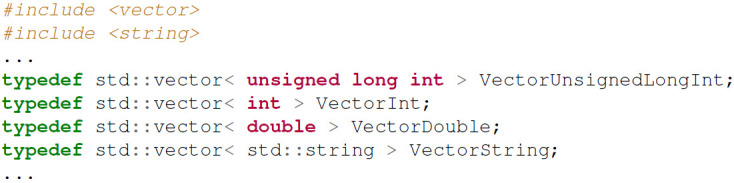



After the generation and compilation of wrappers (using the same procedure as the one described in ‘Wrapping a basic library’), the user can hereafter use C++ containers in the Python interpreter.

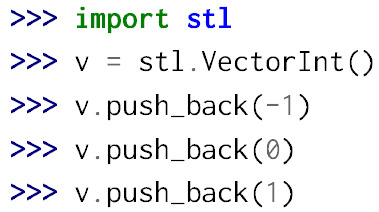



Note that in order to have a functional Python package, some methods can be dynamically added to wrapped classes within modules. For instance, in the stl/vector.py module:

• The __iter__ method that enables iterations over a wrapped vector and its conversion to Python list is added to all std::vector class template instantiations wrapped. 
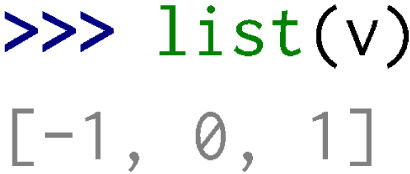

• The __str__ and __repr__ methods that enable representations in the Python interpreter of vectors are added to all std::vector class template instantiations wrapped. 
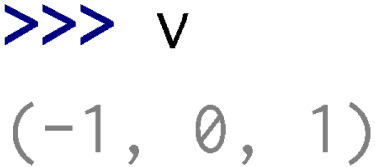

Moreover, the stl/__init__.py module imports all the Python objects of the stl/__stl.so library at its root to simplify class names (e.g., stl.VectorInt instead of stl.__stl.VectorInt).

Some additional features are automatically added in the AutoWIG wrappers. For example, for functions returning non-constant references (e.g., 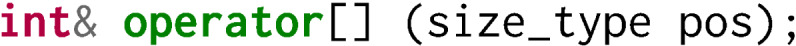 of the std::vector<  int > instantiation), an additional wrapping is done using the following decorator:


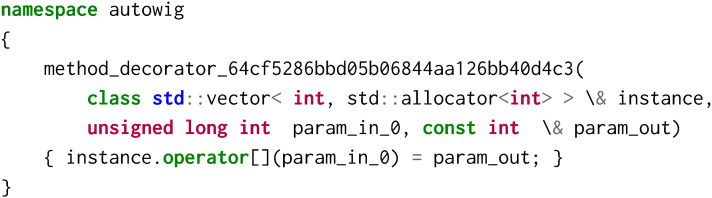


This decorator is then dealt as an overloaded method in wrappers. In this particular example, it enables to define __getitem__ and __setitem__ methods in the stl/vector.py module.

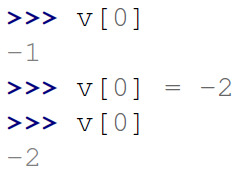



If this decorator is not written, there is no way to use the __setitem__ method in Python. Moreover, since Python users are more familiar with Python containers, each method taking a constant reference or a copy of a C++ container as parameter tries to automatically convert Python objects into the corresponding C++ container. Therefore, as illustrated below, Python list of integers are automatically converted into C++ vectors of integers.

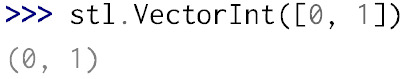



### Wrapping dependent libraries

**Figure 3:**
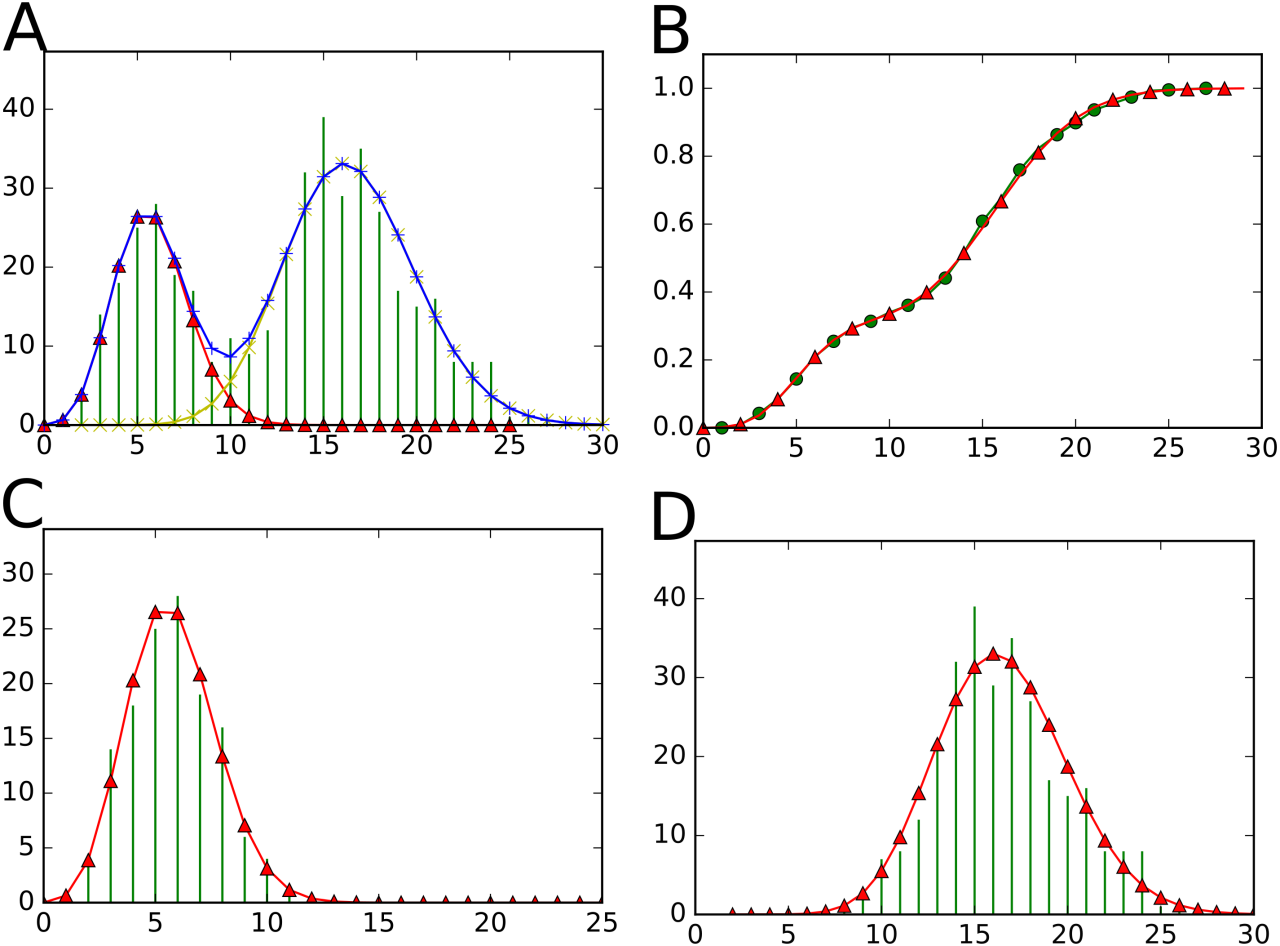
Visualizations proposed by the StatTool Python bindings for mixture model quality assessment. (A) The data frequency distribution is represented in green. The theoretical frequency distribution of the fitted mixture model with two components is represented in blue. (B) The empirical cumulative distribution function is represented in green. The cumulative distribution function of the fitted mixture model with two components is represented in red. (C) (resp. (D)) The empirical probability mass function for the data subset corresponding to the first (resp. second) component is represented in green. The probability mass function of the first (resp. second) component of the fitted mixture model with two components is represented in red.

**Figure 4:**
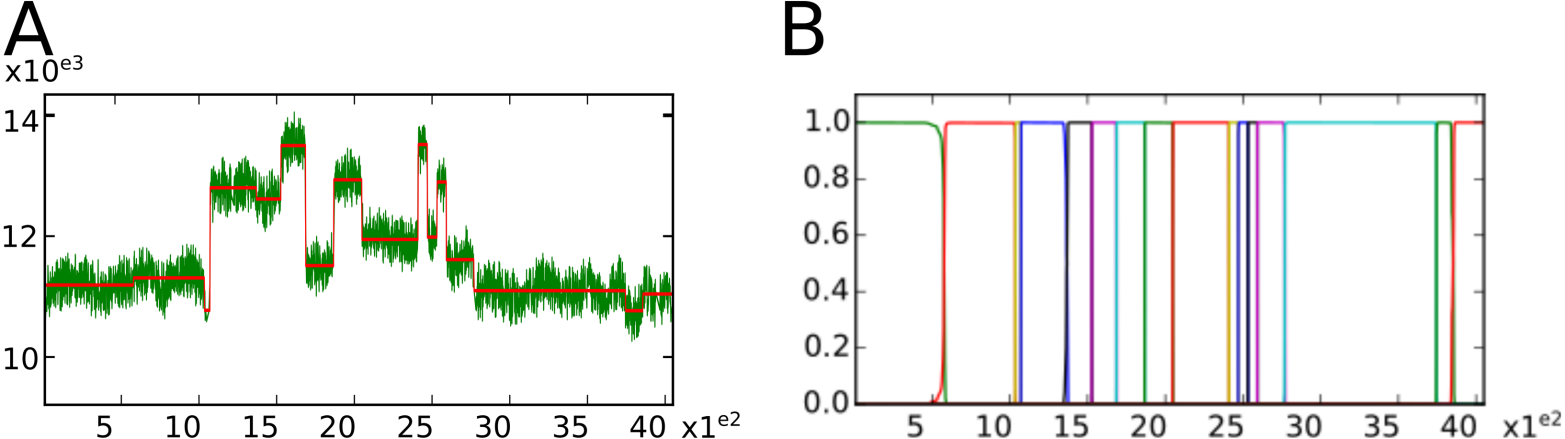
Visualizations proposed by the SequenceAnalysis Python bindings for segmentation quality assessment. (A) In green the nuclear-magnetic response of underground rocks is represented in function of the depth. Segment means are represented by the red piecewise constant function. (B) Posterior segment probabilities.

StructureAnalysis (Guedon & Durand, 2017) is a set of libraries including statistical models for the analysis of structured data (mainly sequences and tree-structured data):

• StatTool is a library containing classes for the parametric modeling of univariate and multivariate data (see Fig. 3).
• SequenceAnalysis is a library containing statistical functions and classes for markovian models (e.g., hidden variable-order Markov and hidden semi-Markov models) and multiple change-point models for sequences (see Fig. 4). The SequenceAnalysis library depends on the StatTool library.

These libraries have been extensively used for the identification and characterization of developmental patterns in plants from the tissular to the whole plant scale. Previously interfaced with AML (a home-made, domain-specific programming language), some work has been done to switch to Python. Nevertheless, the complexity of writing wrappers with Boost.Python limited the number of available components in Python in comparison to AML. One advantage of having a statistical library written in C++ available in Python is that developers can benefit from all other Python packages. As illustrated in Figs. 3–4, this is particularly useful for providing visualizations for statistical model assessment using—for example—the Matplotlib (Hunter, 2007) Python package.

**The StatTool library** In order to wrap a C++ library, that will be used as a dependency by other libraries, the user needs to save the ASG resulting from the wrapping process. In the StatTool case, we first generate the wrappers (using the same procedure as the one described in ‘Wrapping a basic library’). Then, we use the pickle Python package for serializing the StatTool ASG in the ‘ASG.pkl’ file.

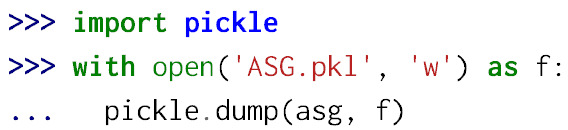



After the compilation of the wrappers, the user can hereafter use mixture models in the Python interpreter. For instance, we considered an example concerning the identification of preformed and neoformed parts in plants.

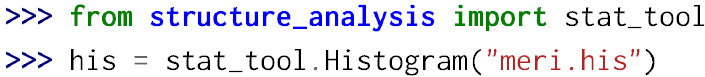



The data ( 
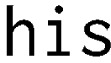
) consists of the number of elongated organs of 424 shoots of wild cherry tree (*Prunus avium*). These shoots were sampled in different architectural positions (from the trunk to peripheral positions of the trees) and were representative of the full range of growth potential. The proximal part of a shoot always consists of preformed organs (i.e., organs contained in the winter bud). This preformed part may be followed by a neoformed part consisting of organs differentiated and elongated during the current growing season. We estimated mixture of parametric discrete distributions on the basis of this data. The number of components (2) was selected between 1 and 4 using the bayesian information criterion.

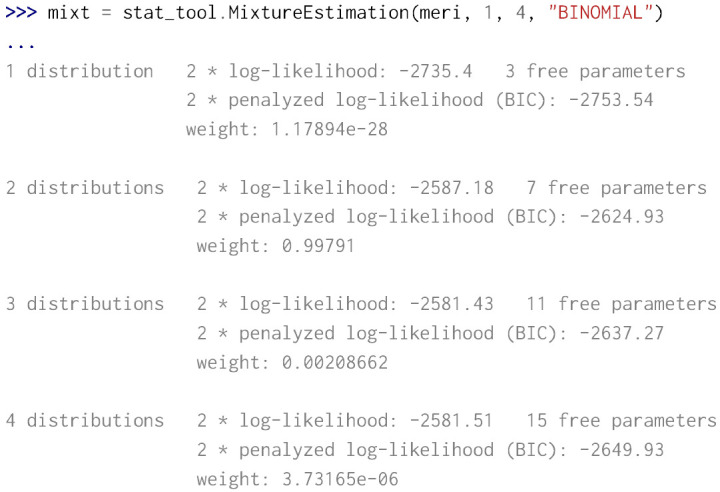



Further investigations can be performed in order to asses the quality of the two component mixture models. For instance, we considered here the visualization of various probability functions.

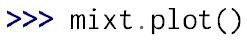



As illustrated on Fig. 3 the data are well fitted by the mixture model and:

• The first component corresponds to entirely preformed shoots.
• The second component to mixed shoots consisting of a preformed part followed by a neoformed part.

**The StructureAnalysis library.** In order to wrap a C++ library that has dependencies, the user needs to combine the ASGs resulting from the wrapping of its dependencies before performing its own wrapping. In the SequenceAnalysis case, we construct first an empty ASG.

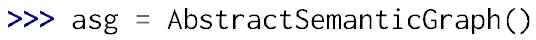



Then, we use the pickle Python package for de-serializing the StatTool ASG (assumed to be serialized in the ‘./stat_tool/ASG.pkl’ file) and merge it with the current ASG.

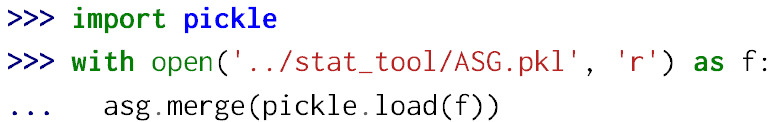



After the generation and compilation of wrappers (using the same procedure as the one described in section ‘Wrapping a basic library’), the user can hereafter use multiple change-point models (see Guédon et al., 2007; Legave et al., 2015 for applications of multiple change-point models) in the Python interpreter. Multiple change-point models are used to delimit segments within sequences, for which the characteristics of variables (or vectors in the multivariate case) are homogeneous within each segment while differing markedly from one segment to another (e.g., piecewise constant mean and variance for a Gaussian change in the mean and variance model). For instance, we considered the classic example of well-log data (Guédon, 2013; Guédon, 2015a; Guédon, 2015b).

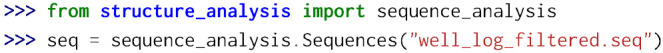



The data ( 
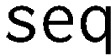
) consist of 4,050 measurements of the nuclear-magnetic response of underground rocks. The data were obtained by lowering a probe into a bore-hole. Measurements were taken at discrete time points by the probe as it was lowered through the hole. The underlying signal is roughly piecewise constant, with each constant segment relating to a single rock type that has constant physical properties. The change points in the signal occur each time a new rock type is encountered. Outliers were removed before the data were analyzed. We estimated Gaussian change in the mean and variance models on the basis of the well-log filtered data. The number of segments (16) was selected using the slope heuristic (Guédon, 2015b) with a slope estimated using log-likelihood of overparametrized models ranging from 30 up to 80 change points.

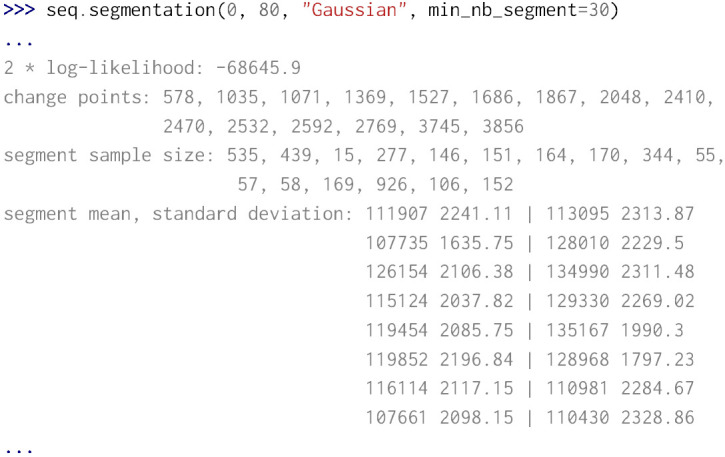



Further investigations can be performed in order to asses the non-ambiguous character of the segmentation into 16 segments. For instance, we considered here the visualization of segment profiles (Guédon, 2013; Guédon, 2015a, see Fig. 4).

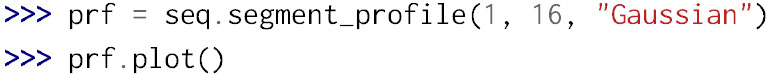



## Discussion

### Related work

Python and R are interpreted languages implemented in C. Like many other scripting languages, they provide a C API (i.e., Application Programming Interface) to allow foreign libraries implemented in C or in a language compatible with C (e.g., C++ or Fortran) to extend the language. This design feature has been a key element for the adoption of the Python language as a glue language, by providing efficient standard libraries implemented in compiled languages. This C API is designed to be stable but low-level. It does not provide support for object-oriented languages, and every type and function has to be *manually* wrapped. Note that although this approach is only efficient when exposing a small number of functions and objects, it is at the basis of all wrapper tools that generate C API code.

Several *semi-automatic* solutions (e.g., Cython, SWIG and Boost.Python) have been proposed to simplify and ease the process of wrapping large C++ libraries. SWIG (Beazley, 2003; Beazley, 2009) implements its own compiler that simplifies the process of wrapping large C and C++ libraries into a large number of different languages, and in particular R and Python. While SWIG is capable of wrapping most of the C++ features, it requires configuration files to include interface and conversion specifications. If there is a change in the library, these configuration files may become out of date. Yet, for basic libraries where all classes should be wrapped SWIG can almost automatically produce wrappers using a concise configuration file. Cython (Behnel et al., 2011) is another semi-automatic solution. Cython both enables Python users to compile Python code to C for optimizing execution of scientific code, and makes it possible for developers to call C or C++ code from Python. Cython is intensively used by several Python scientific libraries (Pedregosa et al., 2011; Van Der Walt et al., 2014) that optimized critical part of their code by writing subparts of the package in Cython. It has been originally developed as part of the Sage project (The Sage Developers, 2015) to integrate numerous packages and libraries written in C, C++ and Fortran. However, Cython requires re-declaration of every class and function to wrap a C or C++ library. Finally, Boost.Python (Abrahams & Grosse-Kunstleve, 2003) and Rcpp (Eddelbuettel et al., 2011) depend on meta-programming to provide high-level abstractions (e.g., registration of classes and inheritance, automatic conversion of registered types and classes, management of smart pointers, C++ object-oriented interface to Python objects, …). However, all the wrappers have to be written and keep in sync with the code of the library, and require lots of knowledge for developers.

Recently, several projects have provided *automatic* solutions for wrapping existing C++ libraries. They mainly rely on the same kind of architecture:

• A parser or compiler that extracts information about the list of C++ functions or classes and their signatures.
• Strategies to convert this abstract view of the C++ code into manual or semi-automatic wrapper tools.
• The generation of the Python or R bindings based on these information.

The first difficulty is to parse large C++ code, and provide information on its structure. For this, tools like Doxygen or GCC-XML have been used. While Doxygen was first developed to automatically extract and render documentation of C++ libraries, it provides an XML representation of the C++ interface that can be used to describe functions and classes. Later, GCC-XML has been developed to offer a representation of a C++ library in XML using the GCC compiler. This tool has been developed for one of the first automatic library, CABLE, used to wrap the large visualization library VTK (Schroeder, Martin & Lorensen, 1997). However, maintaining such a tool is complex and GCC-XML does not support the C++11 standard. In AutoWIG, we use the LLVM/Clang technologies (Lattner, 2008) to have the latest version of the compiler. Clang provides a full representation of the compiled library. Among the automatic tools, CABLE and WrapITK (Lehmann, Pincus & Regrain, 2006) generate SWIG configuration files to build the wrappers, Py++ (Yakovenko, 2011) generates Boost.Python code, and XDress (Scopatz, 2013) generates Cython files. Some domain specific tools, like Shiboken, have also been developed to wrap their large C++ libraries (in this case the entire QT libraries). While these tools provide an excellent solution for very complex libraries, they have some limitations. Some libraries rely on GCC-XML that does not support modern C++ standard. However, a new tool CastXML is currently in development. The main tools depends on configuration files and are called as executable like XDress and WrapITK. While they can easily be integrated in development workflow, it is not easy for developers to drive and specialize them using a scripting language. AutoWIG and Py++ provide a Python interface and offer introspection facilities for C++ libraries from Python. Like Py++, AutoWIG generates Boost.Python wrappers. However, Py++ depends on GCC-XML and requires to write a full parser and code generator in Python. It allows to implement a fully automatic system for developers based on their library design pattern, but is rather complex to implement.

### Extensibility

As stated above, the plugin architecture of AutoWIG enables non-intrusive extensibility. This is of great interest when considering the addition of other source or target languages.

**Figure 5:**
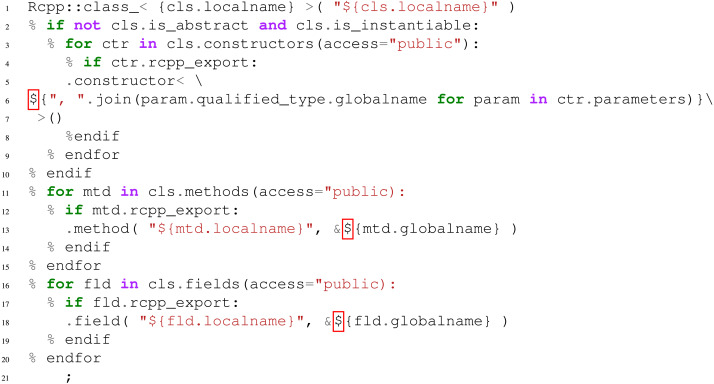
An example of a Mako template used in AutoWIG that would be written to extend AutoWIG to generate R bindings based on Rcpp (Eddelbuettel et al., 2011) for C++ libraries. The cls input of this Mako template must be a node of the abstract semantic graph representing a C++ class.

The addition of a target language principally consists in writing Mako templates (Bayer, 2012). As an example, let consider the R language. In order to be able to propose automatic R bindings for C++ libraries, the templates written could be based on the Rcpp (Eddelbuettel et al., 2011) library. This is particularly interesting since Rcpp wrappers are quite similar to Boost.Python ones (c.f., Fig. 5). As a matter of fact, the implementation of a r_cpp generator is of highest priority regarding future work. The major difficulty encountered is the lack of some features in Rcpp (e.g., enumeration wrapping) and particular organization of R packages that must be taken into account.

The addition of a source language is more problematic since it could lead to addition of new proxy classes in the abstract semantic graph. For example, if the addition of the C, Objective C or Objective C++ languages should be relatively easy since it can be done using the Clang parser and C++ proxy classes, the addition of the Fortran language requires more work. In fact, for this purpose the Open Fortran Parser (Rasmussen & Sottile, 2012) could be used but it would require to reimplement the transformation of an abstract syntax tree to an abstract semantic graph. Moreover, any addition of a source language must be followed with the addition of target language generator since wrapper technologies are dependent of source languages. For Fortran, once the parser is implemented, this would require in addition to implement a generator potentially based on the F2Py (Peterson, 2009) tool.

### Toward a reference guide generator

In its current stage, AutoWIG translates the Doxygen (Van Heesch, 2008) documentation into a Sphinx one (Brandl, 2009) but only incorporates it in the wrappers. This means that both Doxygen and Sphinx tools must be used to respectively generate C++ and Python reference guides. Writing a generator that would generate Sphinx compatible files containing the C++ reference guide could be of great interest since it would allow to aggregate both C++ and Python documentation within the same tool.

### Installation and usage

The installation of AutoWIG has been tested on 64 bits Linux, MacOs X and Windows with Python 2.7 and 3.6. Note that the wrappers generated by AutoWIG do not depend on AutoWIG and can be built on other operating systems (e.g., MacOsX, Windows) than the one used to generate the wrappers (e.g., Linux). On each of these operating systems, AutoWIG binaries are available using the Conda package management system. Note that these binaries require to be installed in a specific environment that will be used for wrapper generation but not for compiling these wrappers since conflicts can occur between AutoWIG’s requirements and those of the wrapped library.

Moreover, examples presented herein can be replayed using the Jupyter notebook (Perez & Granger, 2007) from Docker images (Merkel, 2014) (see supplementary materials). Note that the StructureAnalysis example cannot be played with Python 3.6 nor in Windows since:

• The C++ libraries are not yet compatible with Windows.
• The Python interfaces based on generated wrappers are not yet compatible with Python 3.6.

## Concluding Remarks

AutoWIG greatly simplifies the process of incorporation of compiled libraries within scripting language interpreter. It provides the concept of ASG as C++ code abstraction data model. AutoWIG can therefore be used for C++ code introspection in a Python interpreter to discover and analyze C++ library components. This enabled us to propose an automatic generation of Python bindings for C++ libraries respecting some guidelines (see Table 3). This generation of Python bindings is also combined with the automatic generation of pythonic interface (e.g., use of special functions, error translation, memory management and Sphinx formatted documentation) using Mako, a template language classically used in web frameworks. Some compilation problems led us to also to consider a tool for parsing compiler errors that is particularly useful when considering the wrapping of class template specializations.

**Table 3:** Quantitative summary of the wrapping processes executed in examples. SLOC is the acronym for Source Lines of Code (computed only for parsed headers). Most of non-wrapped functions or variables are C++ components that do not belong the actual C++ library but that are used internally.

C++ Library	Headers		Classes		Functions		Variables	
Example	Parsed	SLOC	Parsed	Wrapped	Parsed	Wrapped	Parsed	Wrapped
Basic library	3	290	4	4	15	10	1	1
ClangLite	687	292,907	2,202	141	20,416	2,061	2,488	18
STL	42	34,519	110	17	679	133	39	1
StructureAnalysis	644	175,603	2,591	116	6,869	1,386	1,758	361

Note that a particular attention has been payed for the AutoWIG architecture:

• It has been designed as a library. This choice has been made since it enables interactive wrapping of compiled libraries in Python. An user can use of AutoWIG interactively to supervise or debug the wrapping process and reduces the level of expertise required to use this software.
• It has been designed as a plugin-oriented architecture. This choice has been made for extensibility purpose to enhance the adoption of AutoWIG by developers. The plugin architecture simplify the integration process of external contribution. While only C++ to Python bindings have been implemented, future work will consider adding new source (such as C) and target (such as R) languages using the plugin architecture.

In ‘Results’, we demonstrated the efficiency of using AutoWIG to wrap large and complex C++ libraries, such as Clang. Such an approach can be used to wrap other very large scientific libraries in an automatic way and enhance their diffusion to large communities of scientists that only use high-level scripting languages such as Python and R.

Considering development teams, it is possible to incorporates AutoWIG with software construction tools to reduce the level of expertise required to generate wrappers. For now, AutoWIG provides a SCons tool working with Conda and SCons and that automates the most common wrapping steps. For an example, the reader can refer to the STL repository (http://github.com/StatisKit/STL), within which this tool is used.

## Additional Information and Declarations

### Data Availability

The following information was supplied regarding data availability: The source code of AutoWIG is available on GitHub (http://github.com/StatisKit/AutoWIG; https://github.com/StatisKit/AutoWIG/releases/tag/v1.0.0). The documentation is hosted on Read The Docs (http://autowig.rtfd.io). The new Python interface to the Clang library and the AutoWIG parser implemented using this interface are available on GitHub (http://github.com/StatisKit/ClangLite). Examples presented in this article are available on GitHub (http://github.com/StatisKit/FP17; https://github.com/StatisKit/FP17/releases/tag/v1.0.0) and reproducible using Docker images as presented in the README.rst (https://hub.docker.com/r/statiskit/fp17/tags/).
